# Mentalizing Failures, Emotional Dysregulation, and Cognitive Distortions Among Adolescent Problem Gamblers

**DOI:** 10.1007/s10899-020-09967-w

**Published:** 2020-07-27

**Authors:** Maria Ciccarelli, Giovanna Nigro, Francesca D’Olimpio, Mark D. Griffiths, Marina Cosenza

**Affiliations:** 1 Department of Psychology, Università degli Studi della Campania “Luigi Vanvitelli”, Viale Ellittico, 31, 81100 Caserta, Italy; 2 Psychology Department, Nottingham Trent University, Shakespeare Street, Nottingham, NG1 4FQ UK

**Keywords:** Gambling, Problem gambling, Adolescence, Mentalization, Emotional dysregulation, Cognitive distortions

## Abstract

Over the past decade, several studies have investigated the relationship between cognitive distortions and emotion regulation among adolescent gamblers, demonstrating the exacerbating role of alcohol consumption when co-occurring with gambling problems. An important construct, that to date has been largely neglected, is mentalizing (i.e. the ability to reflect on one's own and others' mental states). The aim of the present study was (for the first time) to investigate the relative contribution of mentalization, emotional dysregulation, cognitive distortions, and alcohol consumption among adolescent gamblers. A total of 396 students (69.2% females) aged 14–19 years were recruited from secondary schools in Southern Italy. Assessment measures included the South Oaks Gambling Screen Revised for Adolescents (SOGS-RA), the Reflective Functioning Questionnaire (RFQ-8), the Difficulties in Emotion Regulation Scale (DERS), the Gambling Related Cognitions Scale (GRCS), and the Alcohol Use Disorders Identification Test (AUDIT). Regression analysis showed that, along with male gender, the best predictors of adolescent gambling were scores on two GRCS subscales (i.e., ‘inability to stop gambling’ and ‘interpretative bias’), the RFQ-8’s ‘uncertainty about mental states’ dimension, and the DERS ‘impulse control difficulties’ factor, with the overall model explaining more than one-third of the total variance. The results clearly indicated that, along with gambling-related cognitive distortions, uncertainty about mental states, and difficulties remaining in control of one’s behavior when experiencing negative emotions contributed significantly to problematic gambling among adolescents.

## Introduction

A systematic review of the most recent international studies published (2000–2016) highlighted that European adolescent problem gambling prevalence rates ranged from 0.2 to 5.6% (Calado et al. 2017). In Italy (where the present study was carried out), a national 2018 survey by the Italian National Institute of Health among young students aged between 14 and 17 years reported that about 30% of minors had gambled at least once in the previous year, and that 10% of them were problem gamblers (Istituto Superiore di Sanità 2018). The continuous growth of gambling opportunities and the easy accessibility to many forms of gambling has resulted in a significant increase in the prevalence of adolescent problem gambling (Andrie et al. 2019; Calado et al. 2017; Dei et al. 2020; Delfabbro et al. 2016). As numerous studies have highlighted, an early age of gambling onset is associated with a higher probability of developing gambling problems (e.g. Carbonneau et al. 2015; Griffiths 1995; Potenza et al. 2011; Rahman et al. 2012), engaging in other risk behaviors (Lynch et al. 2004; Welte et al. 2009), and experiencing mental disorders (Grant et al. 2009). In the light of these findings, understanding the factors characterizing adolescent problem gambling is crucial in developing intervention strategies aimed at preventing the development of addiction, given that this age group is more susceptible to problem gambling than adults (Oh et al. 2017; Wilber and Potenza 2006).

Mentalizing is an underexplored construct in adolescent problem gambling. It refers to the ability in reflecting on self and others’ internal mental states such as feelings, attitudes, goals, beliefs, and emotions (Fonagy et al. 2012). Mentalizing impairments can be of two types: *hypomentalizing* and *hypermentalizing* (Fonagy and Bateman 2016). Whereas hypomentalizing reflects the inability to consider complex models of one’s own and others’ mind, hypermentalizing consists in complex models of mind that currently have no empirical evidence. Temporary or stable failures in mentalization are involved in a wide array of psychopathology, including depression (Luyten et al. 2012), anxiety (Spada et al. 2008a), and personality disorders (Fonagy et al. 2016). Furthermore, mentalization deficits have been found to predict several addictive behaviors including gambling (Möller et al. 2016; Spada and Roarty 2015; Spada et al. 2007, 2008b, 2009). To date, only one study has investigated the role of reflective functioning among adolescent gamblers, highlighting that general impairments in mentalizing play a key role in adolescent problematic gambling, and that, more specifically, hypomentalizing mediates the relationship between dysfunctional impulsivity and gambling involvement (Cosenza et al. 2019b).

Mentalizing impairments are strongly associated with difficulties in emotion regulation (e.g., Sharp et al. 2011). More specifically, difficulties with impulse control, the lack of emotional competence, and the tendency to use maladaptive emotion regulation strategies positively contribute to adult problem gambling (Elmas et al. 2017; Marchica et al. 2019b, 2020; Navas et al. 2017; Williams et al. 2012). Deficits in emotion regulation predispose individuals to experience negative emotions (Jauregui et al. 2016; Veilleux et al. 2014), as documented by high levels of anxiety and depression found among disordered gamblers (e.g. Cosenza et al. 2019a; Nigro et al. 2017, 2019a, b). Although previous studies have observed significant associations between adolescent problem gambling, alexithymia (Cosenza et al. 2014), and negative emotions (Cosenza et al. 2019a; Nigro et al. 2017), only one study (Estévez et al. 2017) has investigated emotional dysregulation in non-substance-related addictions among adolescents, demonstrating that emotion dysregulation predicts several addictive behaviors, including gambling.

A recent cognitive conceptualization of gambling has highlighted important relationships among emotions, cognitions, and gambling, suggesting that the association between negative emotions and gambling severity is mediated by irrational thinking about gambling (Raylu et al. 2016). The importance of cognitive distortions in gambling was initially brought to light by Gaboury and Ladouceur (1989) who observed that the majority of the participants’ verbalizations during a gambling session were irrational. Some of these gambling-related cognitive distortions concern the illusion of controlling gambling outcomes utilizing specific behaviors or rituals (i.e. illusion of control), the tendency to selectively remember wins (i.e. interpretative bias), and to make predictions about gambling outcomes based on previous experience (i.e. predictive control) (Ciccarelli et al. 2017; Fortune and Goodie 2012; Griffiths 1994; Ledgerwood et al. 2019; Mallorquí-Bagué et al. 2019; Parke et al. 2007).

In the light of these data, the first aim of the present study was to investigate more deeply two underexplored constructs in adolescent gambling: mentalization and emotional dysregulation. The second aim was to evaluate, for the first time, the interplay between mentalization, emotional dysregulation, and irrational beliefs in adolescent gambling. Given the literature highlighted the frequent association of adolescent gambling behavior with alcohol consumption (e.g. Ciccarelli et al. 2019a, b; Ciccarelli et al. 2020; Dowling et al. 2017; Leino et al. 2017; Tobias-Webb et al. 2019; Wilber and Potenza 2006), the study controlled for alcohol use.

## Methods

### Participants and Procedure

The sample comprised 396 students (69.2% females), aged between 14 and 19 years (*M*_age_ = 17.22 years; *SD* = 1.03), attending from the first-to the fifth-year of six public high schools (lyceums) in Southern Italy (cities of Naples and Salerno). No-one at the randomly selected schools declined to participate. The only inclusion criterion was having gambled at least once in the last year. Data collection began after the approval of the study protocol by the institutional review boards, from March to April 2019. Informed consent from the head of the school and from participants, or from their parents if minors, was obtained prior to data collection. The questionnaires were completed in the classroom during school hours.

Participants completed the Italian versions of the South Oaks Gambling Screen-Revised for Adolescents (SOGS-RA; Winters et al. 1993, 1995; Colasante et al. 2014), the Gambling Related Cognitions Scale (GRCS; Raylu and Oei 2004; Donati et al. 2015), the Difficulties in Emotion Regulation Scale (DERS; Gratz and Roemer 2004; Sighinolfi et al. 2010), the Reflective Functioning Questionnaire (RFQ-8; Fonagy et al. 2016; Morandotti et al. 2018), and the Alcohol Use Disorders Identification Test (AUDIT; Saunders et al. 1993; Piccinelli et al. 1997). The order of presentation of measures was counterbalanced. Anonymity and confidentiality were guaranteed to all participants. For each measure, participants received written instructions. Administration of the survey took between 20 and 25 min to complete. Ethics approval was obtained from the research team’s university Psychology Department ethics committee.

### Measures

The SOGS-RA is a self-report instrument that comprises 12 dichotomous (*yes/no*) scored items related to gambling behavior over the past 12 months and several non-scored items concerning the amount of money gambled, the gambling motivations, and the frequency of participation in gambling activities (cards, horses, casinos, etc.). The scores range from 0 to 12, where higher scores indicate greater gambling severity. More specifically, adolescents who score 0 to 1 are classified as non-problem gamblers, whereas those who score 2–3 are classified as at-risk gamblers, and those who score 4 or above are classified as problem gamblers. The SOGS-RA in the present study was found to have adequate internal consistency reliability coefficient (Cronbach’s α = 0.65; 95% CI [0.58–0.71]).

The RFQ-8 is a self-report scale assessing the reflective functioning. It comprises eight items that concern two different processes: ‘certainty about mental states’ and ‘uncertainty about mental states’. Participants are required to indicate how much they disagree or agree with each statement using a seven-point Likert scale, from “strongly disagree” to “strongly agree”. In the certainty scale, low agreement indicates excessive but inaccurate mentalizing (*hypermentalizing*), while high agreement indicates more genuine mentalizing. In the uncertainty scale, high agreement reflects lack of knowledge about mental states (*hypomentalizing*), while low agreement reflects acknowledgment of the opaqueness of one’s own mental states and that of others, characteristic of genuine mentalizing. Cronbach’s α for the full scale was 0.61 (95% CI [0.55–0.67]) and 0.61, (95% CI [0.55–0.67]) for the uncertainty subscale, and 0.68 (95% CI [0.63–0.72]) for the certainty subscale.

The DERS is a 36-item self-report instrument assessing several dimensions of emotional dysregulation comprising six scales: non-acceptance of emotional responses (e.g., “When I’m upset, I feel like I am weak”), difficulties engaging in goal-directed behavior (e.g., “When I’m upset, I have difficulty getting work done”), impulse control difficulties (e.g., “I experience my emotions as overwhelming and out of control”), lack of emotional awareness (e.g., “I am attentive to my feelings”, reverse coded), limited access to emotion regulation strategies (e.g., “When I’m upset, I start to feel very bad about myself”), and lack of emotional clarity (e.g., “I am confused about how I feel”). Participants have to indicate how much each item describes themselves using a five-point Likert scale from “almost never” to “almost always”. Higher scores are reflective of greater problems with emotion regulation. In the present study, Cronbach’s α for the full scale was 0.91 (95% CI [0.90–0.92]) and 0.85, (95% CI [0.82–0.87]) for non-acceptance of emotional responses, 0.84 (95% CI [0.82–0.87]) for difficulties engaging in goal-directed behavior, 0.83 (95% CI [0.80–0.85]) for impulse control difficulties, 0.70 (95% CI [0.65–0.74]) for lack of emotional awareness, 0.86 (95% CI [0.84–0.88]) for limited access to emotion regulation strategies, and 0.80 (95% CI [0.77–0.83]) for lack of emotional clarity.

The GRCS assesses the extent to which individuals hold common gambling distortions. It comprises 23 items that specifically identify five cognitive domains: gambling-related expectancies (e.g., “Gambling makes things seem better”), illusion of control (e.g., “I have specific rituals and behaviours that increase my chances of winning”), predictive control (e.g., “Losses when gambling are bound to be followed by a series of wins”), perceived inability to stop gambling (e.g., “I’m not strong enough to stop gambling”), and interpretive bias (e.g., “Relating my winnings to my skill and ability makes me continue gambling”). Individuals indicate the extent to which they agree with each statement on a seven-point scale ranging from “strongly disagree” to “strongly agree”. High scores reflect high levels of irrational beliefs. In the present study, internal consistency for the total scale (α = 0.90, 95% CI [0.89–0.92]) and for each scale was adequate in the present sample: gambling-related expectancies (α = 0.71, 95% CI [0.66–0.76]), illusion of control (α = 0.69, 95% CI [0.63–0.73]), predictive control (α = 0.75, 95% CI [0.71–0.78]), perceived inability to stop gambling (α = 0.59, 95% CI [0.52–0.65]), and interpretative bias (α = 0.76, 95% CI [0.72–0.80]).

The AUDIT is a 10-item self-report tool that assesses the severity of alcohol intake responded to on a five-point scale from “never” to “daily or almost daily”. A score of 8 or more indicates a strong likelihood of harmful alcohol use. In the present study, Cronbach’s α was very good (0.80; 95% CI [0.76–0.82]).

### Data Analysis

The SPSS-20 program was used to analyze the data with a significance level of *p* < 0.05. Pearson correlation coefficients were run to examine the relationships between the SOGS-RA, RFQ-8, DERS, GRCS, and AUDIT. Descriptive statistics were used to describe the main characteristics of the sample. Chi-square analyses were undertaken to examine gender distribution and differences in gambling habits among gambling groups. For the quantitative variables, analysis of variance (ANOVA) was used. To identify predictors of adolescent problem gambling, a linear regression analysis was performed with SOGS-RA total score as the dependent variable, and gender, RFQ-8, DERS, GRCS subscales, and AUDIT total score as independent variables.

## Results

All variables were initially screened for missing data, distribution abnormalities, and outliers (Tabachnick and Fidell 2013). Missing data (< 3%) were replaced with means. Since the distribution of the SOGS-RA was positively skewed, square-root transformation was performed and used for the analysis. Consequently, the assumptions of normality, linearity, and homoscedasticity were adequately met.

### Correlational Analysis

Correlational analysis showed positive associations of SOGS-RA score with male gender (r = − 0.173; *p* = 0.001), and scores on the RFQ-8, DERS, GRCS subscales, and AUDIT. The SOGS-RA score also correlated with some DERS subscale scores (i.e., non-acceptance of emotional responses, difficulties engaging in goal-directed behavior, impulse control difficulties, and limited access to emotion regulation strategies). Correlations between uncertainty (RFQ-8), AUDIT, and both GRCS and DERS dimensions were found (see Table 1).

**Table 1:** Pearson correlation coefficients of measures of interest among adolescent gamblers

	1	2	3	4	5	6	7	8	9	10	11	12	13	14
1. SOGS-RA	–													
2. RFQ-8-CERTAINTY	− .186**	–												
3. RFQ-8-UNCERTAINTY	.272**	− .569**	**–**											
4. DERS-NONACC	.177**	− .348**	.349**	–										
5. DERS-GOALS	.207**	− .254**	.390**	.400**	–									
6. DERS-IMPULSE	.268**	− .307**	.461**	.364**	.536**	**–**								
7. DERS-AWARENESS	.040	− .150**	.087	**− **.023	**− **.088	.005	–							
8. DERS-STRATEGIES	.182**	− .286**	.385**	.655**	.617**	.546**	**− **.072	–						
9. DERS-CLARITY	.119	− .334**	.467**	.422**	.343**	.361**	.262**	.502**	–					
10. GRCS-GE	.412**	− .129*	.109*	.038	.047	.133**	.069	.041	**− **.008	–				
11. GRCS-IC	.340**	− .170**	.170**	.056	**− **.004	.096	.036	.018	.052	.489**	**–**			
12. GRCS-PC	.443**	− .173**	.232**	.038	.035	.128*	.092	**− **.018	**− **.018	.571**	.651**	–		
13. GRCS-IS	.482**	− .183**	.156**	.079	.083	.224**	.040	.076	.002	.618**	.396**	.538**	–	
14. GRCS-IB	.513**	− .158**	.183**	.062	.108*	.176**	.068	.042	**− **.011	.635**	.562**	.732**	.624**	–
15. AUDIT	.159**	− .145**	.222**	.166**	.174**	.320**	**− **.047	.289**	.193**	.126*	.098	.056	.083	.066

Bold values represent significant correlation coefficients

*SOGS-RA* South Oaks Gambling Screen-Revised for Adolescents, *DERS-NONACC* Non-acceptance of emotional responses, *DERS-GOALS* Difficulties engaging in goal-directed behavior, *DERS-IMPULSE* Impulse control difficulties, *DERS-AWARENESS* Lack of emotional awareness, *DERS-STRATEGIES* Limited access to emotion regulation strategies, *DERS-CLARITY* Lack of emotional clarity, *GRCS-GE* Gambling expectances, *GRCS-IC* Illusion of control, *GRCS-PC* Predictive control, *GRCS-IS* Inability to stop, *GRCS-IB* Interpretative bias

*p < 0.05; **p < 0.01

### Differences Between Gambling Groups

Based on the SOGS-RA scores, 78.8% of participants were classed as non-problem gamblers, 17.9% as at-risk gamblers, and 3.3% as problem gamblers. Since no significant differences were found in mentalizing, emotion regulation, cognitive distortions, and alcohol intake between at-risk gamblers and problem gamblers, they were combined into a single group of “at-risk/problem gamblers”, in line with previous studies (e.g., Blinn-Pike et al. 2010; Ciccarelli et al. 2016a, b; Lee et al. 2011). Chi-square analyses showed differences in the distribution of male and female participants among SOGS-RA groups, [χ^2^(1) = 12.20; *p* = 0.001], with non-problem gambling group comprising mainly of females. No age differences were found in relation to gambling severity (*F*_1,394_ = 0.14; *p* = 0.71).

Chi-square analysis, run to ascertain the motivations for gambling as function of gambling severity, showed that at-risk/problem gamblers gambled significantly more to win money [χ^2^(1) = 40.19; *p* < 0.001], for entertainment [χ^2^(1) = 24.82; *p* < 0.001], as a hobby [χ^2^(1) = 9.35; *p* < 0.01] and for excitement [χ^2^ (1) = 9.35; *p* < 0.01]. At-risk/problem gamblers gambled mainly in tobacco stores [χ^2^(1) = 6.79; *p* < 0.01], gambling venues [χ^2^(1) = 24.04; *p* < 0.001], and bingo halls [χ^2^(1) = 9.23; *p* < 0.01]. As regard who they gambled with, at-risk/problem gamblers mainly gambled with friends [χ^2^(1) = 24.33; *p* < 0.001], grandparents [χ^2^(1) = 5.85; *p* < 0.05], and alone [χ^2^(1) = 11.46; *p* = 0.001]. The percentages of online and offline gambling activities as a function of the relative frequency of participation during the past twelve months are reported in Table 2. Regarding the amount of money gambled, 54.8% of gamblers had gambled €1–5, 19.1% gambled €5–10, 4.8% gambled €10–50, and 1% gambled €50–100, with at-risk/problem gamblers having gambled more money than the other group [χ^2^(5) = 54.53; *p* < 0.001]. Just over a quarter of participants (27%) reported an age of gambling onset before 11 years of age.

**Table 2:** Percentages of common gambling activities as a function of frequency (12-months-prevalence)

	Never		Less than monthly		Monthly		Weekly		Daily	
	Offline	Online	Offline	Online	Offline	Online	Offline	Online	Offline	Online
Cards	52.5	95.7	32.4	1.8	9.0	1.8	3.0	0.3	2.5	0.0
Horse races	97.0	98.0	1.5	0.8	0.8	0.3	0.3	0.5	0.0	0.0
Sports betting	68.6	88.4	15.1	4.3	5.8	2.0	7.8	3.0	2.3	1.8
Dice	87.2	99.5	10.8	0.0	0.8	0.0	0.0	0.0	0.5	0.0
Casino	98.2	99.0	0.8	0.3	0.3	0.0	0.0	0.0	0.3	0.3
Scratch cards	64.3	97.5	27.9	1.3	6.3	0.5	0.8	0.3	0.3	0.0
Lotteries	82.9	98.5	13.6	0.8	2.5	0.3	0.5	0.0	0.0	0.0
Bingo	88.9	98.5	8.5	0.5	1.8	0.3	0.3	0.3	0.0	0.0
Slot machines	95.2	98.2	3.0	0.3	0.8	0.3	0.0	0.3	0.5	0.5
Skill games	73.6	97.5	15.8	1.3	6.8	0.5	2.0	0.3	1.3	0.0

The ANCOVA executed on AUDIT showed that SOGS-RA groups differed significantly in alcohol consumption (*F*_1,393_ = 10.48; *p* = 0.001; η^2^_*p*_ = 0.03), with a significant effect of gender as a covariate (*F*_1,393_ = 4.07; *p* < 0.05; η^2^_*p*_ = 0.01). At-risk/problem gamblers reported greater problematic alcohol consumption than non-problem gamblers (*p* < 0.05), with males scoring higher than females. For the subsequent analyses, both gender and AUDIT were used as covariates. Table 3 summarizes the test scores of SOGS-RA groups.

**Table 3:** Mean and standard deviations of the data between SOGS-RA groups

	Non-problem gamblers (N = 312)		At-risk/problem gamblers (N = 84)	
	M	SD	M	SD
RFQ-8-CERTAINTY	1.02	0.71	0.71	0.63
RFQ-8-UNCERTAINTY	0.64	0.49	0.92	0.62
DERS-NONACC	12.23	5.40	13.76	5.89
DERS-GOALS	14.99	4.88	16.89	4.82
DERS-IMPULSE	13.06	5.02	15.59	5.51
DERS-AWARENESS	15.69	4.41	15.81	4.08
DERS-STRATEGIES	17.40	6.66	19.12	7.42
DERS-CLARITY	12.51	4.35	13.08	4.45
GRCS-GE	6.02	3.10	8.83	4.03
GRCS-IC	5.71	3.24	8.17	3.97
GRCS-PC	11.06	5.60	16.83	6.59
GRCS-IS	6.61	2.53	9.58	4.21
GRCS-IB	6.59	3.67	11.43	5.11
AUDIT	3.52	3.32	5.08	4.12

*DERS-NONACC* Non-acceptance of emotional responses, *DERS-GOALS* Difficulties engaging in goal-directed behavior, *DERS-IMPULSE* Impulse control difficulties, *DERS-AWARENESS* Lack of emotional awareness, *DERS-STRATEGIES* Limited access to emotion regulation strategies, *DERS-CLARITY* Lack of emotional clarity, *GRCS-GE* Gambling expectances, *GRCS-IC* Illusion of control, *GRCS-PC* Predictive control, *GRCS-IS* Inability to stop, *GRCS-IB* Interpretative bias

To compare SOGS-RA groups on mentalizing, a repeated measure ANCOVA was performed, with group as a between-participants factor, RFQ-8 subscales as dependent variables, and gender and AUDIT score as covariates. The ANCOVA showed significant interactions of RFQ with gender (*F*_1,392_ = 6.27; *p* = 0.01; η^2^_*p*_ = 0.02), AUDIT (*F*_1,392_ = 13.22; *p* < 0.001; η^2^_*p*_ = 0.03) and SOGS-RA (*F*_1,392_ = 17.61; *p* < 0.001; η^2^_*p*_ = 0.04), whereas no main effects of SOGS-RA (*F*_1,392_ = 0.10; *p* = 0.76), AUDIT (*F*_1,392_ = 0.41; *p* = 0.52), and gender (*F*_1,392_ = 0.17; *p* = 0.68) were found. These results demonstrate that, compared to the non-problem gambling group, at-risk/problem gamblers reported higher scores on uncertainty and lower scores on certainty, and females scored higher than males on uncertainty. Furthermore, alcohol consumption correlated with hypomentalizing.

The same ANCOVA run on the DERS subscales yielded main effects of SOGS-RA (*F*_1,392_ = 6.44; *p* = 0.01; η^2^_*p*_ = 0.02) and AUDIT (*F*_1,392_ = 31.03; *p* < 0.001; η^2^_*p*_ = 0.07), but no effect of gender (*F*_1,392_ = 2.27; *p* = 0.13). With regard to interaction effects, a significant DERS x AUDIT effect (*F*_5,388_ = 9.22; *p* < 0.001; η^2^_*p*_ = 0.11) emerged, whereas the interaction of DERS with SOGS-RA (*F*_5,388_ = 1.23; *p* = 0.29) and gender (*F*_5,388_ = 2.14; *p* = 0.06; η^2^_*p*_ = 0.03) were not significant. The results indicated that at-risk/problem gamblers significantly differed from non-problem gamblers on non-acceptance, goals, impulse, and strategies (all *p *values ≤ 0.05). Moreover, the greater the alcohol intake the greater the emotional dysregulation.

With regard to GRCS subscales, the ANCOVA showed main effects of group (*F*_1,392_ = 81.57; *p* < 0.001; η^2^_*p*_ = 0.17) and gender (*F*_1,392_ = 16.20; *p* < 0.001; η^2^_*p*_ = 0.04), and interaction effects of GRCS with both gender (*F*_4,389_ = 7.33; *p* < 0.001; η^2^_*p*_ = 0.07) and SOGS-RA groups (*F*_4,389_ = 11.71; *p* < 0.001; η^2^_*p*_ = 0.11) emerged. No effects of AUDIT (*F*_1,392_ = 0.00; *p* = 0.99) or GRCS x AUDIT (*F*_4,389_ = 1.00; *p* = 0.41) interaction were observed. At-risk/problem gamblers scored significantly higher on all GRCS subscales compared to non-problem gambling counterparts (all *p *values ≤ 0.001). The significant effect of gender indicated that males scored higher than females on all GRCS subscales (*p* < 0.001), except for illusion of control.

### Regression Analysis

To evaluate the contributions of gender, age, mentalizing deficits, emotional dysregulation, cognitive distortions, and alcohol consumption to adolescent gambling severity, a hierarchical linear regression analysis was conducted, using SOGS-RA square-root score as the criterion variable. Along with male gender, interpretative bias and inability to stop (GRCS), uncertainty (RFQ-8), and impulse (DERS) emerged as significant predictors of problematic gambling, with the overall model explaining more than one-third (34%) of the total variance (R^2^*adj* = 0.34; *F*_5,390_ = 42.00; *p* < 0.001) (see Table 4).

**Table 4:** Results of hierarchical linear regression analysis on adolescent problem gambling (SOGS-RA)

Predictors	B	SE	β	*t*	*p* value
*Model 1 (R*^*2*^*adj = 0.03; p < 0.001)*					
Gender	**− **.150	.041	**− **.182	**− **3.645	.000
*Model 2 (R*^*2*^*adj = 0.27; p < 0.001*)					
Gender	**− **.048	.037	**− **.059	**− **1.229	.188
GRCS-IB	.043	.004	.504	11.363	.000
*Model 3 (R*^*2*^*adj = 0.31; p < 0.001)*					
Gender	**− **.070	.036	**− **.085	**− **1.840	.052
GRCS-IB	.039	.004	.460	10.438	.000
RFQ-U	.146	.031	.204	4.771	.000
*Model 4* (R^2^*adj* = 0.33; *p* < 0.001)					
Gender	**− **.047	.036	**− **.057	**− **1.198	.185
GRCS-IB	.028	.005	.328	6.181	.000
RFQ-U	.136	.030	.190	4.553	.000
GRCS-IS	.027	.006	.226	4.306	.000
*Model 5* (R^2^*adj* = 0.34; *p* < 0.001)					
Gender	**− **.041	.036	**− **.049	**− **1.055	.255
GRCS-IB	.028	.005	.329	6.229	.000
RFQ-U	.102	.034	.142	3.110	.003
GRCS-IS	.025	.006	.211	4.003	.000
DERS-IMPULSE	.008	.003	.107	2.121	.023

B, unstandardized coefficient; β, standardized regression coefficient. Gender: 0 = Male; 1 = Female

GRCS-IS, Inability to Stop; GRCS-IB, Interpretative Bias; RFQ-U, Uncertainty; DERS-IMPULSE, Impulse Control Difficulties

## Discussion

The results of the present study are in line with previous findings in literature reporting a higher prevalence rate of problem gambling among males, compared to females (Calado et al. 2017; Cosenza et al. 2014; Cosenza and Nigro 2015; Dowling et al. 2017; Hing et al. 2016; Nigro et al. 2017; Volberg et al. 2018). This finding can be explained in relation to the Gender-as-Proxy Hypothesis, according to which gender influences problem gambling both directly and indirectly, through psychological, social and/or physiological characteristics that tend to differ between males and females even in the general population (Nelson et al. 2006).

In the present study, just over a quarter of the participants reported having started gambling before the age of 11 years. This is arguably surprising given that in Italy gambling activities are legally restricted to adults. Moreover, this result is worrying because at-risk and problem gamblers were found to be more frequent among earlier-onset than among later-onset adolescent gamblers. At the same time, early-onset gambling has been found to be predictive of lifetime gambling problems and associated with a more severe gambling profile (Burge et al. 2006; Griffiths 1995; Jimenez-Murcia et al. 2010; Rahman et al. 2012).

The present findings indicated that problematic gambling in adolescence was associated with a specific deficit in mentalizing, namely hypomentalizing. Furthermore, as the regression analysis showed, this inability to interpret human behavior in terms of mental states predicted problem gambling in adolescence, suggesting that an impaired mentalization may represent a risk factor for disordered gambling. This impairment could facilitate gambling involvement undermining insight into their own behavior and decisions. Recent studies have found that problematic gamblers are more confident in their performance despite performing poorer than healthy controls in both gambling (Brevers et al. 2013) and non-gambling tasks (Brevers et al. 2014). The authors, who found no effect of reward/loss sensitivity on the performance overestimation, hypothesized that this could be related to the impairment of their metacognitive abilities/metacognition (Brevers et al. 2013, 2014), which, like mentalizing, belongs to “higher order cognitions” (Fonagy and Bateman 2016, p. 59). In line with these results, Nigro and colleagues found that impairment in mentalizing is a predictor of chasing behavior, confirming that the inability to mentalize could be one of the factors that facilitates chasing propensity (Nigro et al. 2019a, b). In addition, the significant correlations of both hypomentalization and hypermentalization with cognitive distortions observed in the present study appear to confirm the hypothesis that an over-confidence concerning mental states, as well difficulties in reflecting about mental states, may strengthen irrational beliefs and represent risk factors for more severe gambling involvement (Brevers et al. 2013).

At-risk/problem gamblers showed high levels of emotion dysregulation, specifically related to non-acceptance of negative emotions and difficulties in (1) impulse control, (2) adopting goals-oriented behavior, and (3) developing adaptive emotion regulation strategies in the presence of negative emotions. However, when performing regression analysis, only the dimension related to the difficulty in control impulses when experiencing negative emotions predicted problem gambling. It could be that in conditions of emotional distress, individuals lacking of regulatory skills are vulnerable to impulsive actions, such as the perseveration in gambling participation (Tice et al. 2001; see Rogier and Velotti (2018) and Marchica et al. (2019) for reviews). The negative reinforcement provided by the improvement of the emotional state, in turn, predisposes gamblers to impulsive perseveration in gambling activities (Schreiber et al. 2012), exposing them to a greater risk of developing disordered gambling (Stewart and Zack 2008; Stewart et al. 2008). The present results concur with the broad literature on adult gambling and with a previous study that documented the role of emotional dysregulation in predicting adolescent gambling (Estévez et al. 2017), and suggests that adolescents might engage in risky behaviors to prolong positive emotions (Williams and Grisham 2012), and avoid negative feelings (Aldao et al. 2010).

In the present study, two types of cognitive distortions were found to significantly predict adolescent problematic gambling: inability to stop and interpretative biases. The inability to stop gambling refers to the perception of an individual’s helplessness to control and/or stop gambling activities. Interpretative biases represent the core of gambling misconceptions, in as much as it regards the consideration of wins as the result of an individual’s own ability to influence game outcomes and losses as arising from bad luck. Here, gamblers enter a self-deception loop in which the odds of winning appear greater than the possible losses. These results are in line with previous findings among both adult (Ciccarelli et al. 2016a, b; Labrador et al. 2020) and adolescent samples (Ciccarelli et al. 2017; Cosenza and Nigro 2015; Cosenza et al. 2019; Donati et al. 2018) and confirm the role of cognitive distortions in fostering gambling involvement.

Because of the correlational nature of the present study, the results should be interpreted with caution. First, data were exclusively based on self-report measures that limit the generalizability of the results due to recall bias and social desirability. Another limitation is the different group size (non-problem vs. problem gamblers). Future studies should recruit a more consistent group of adolescent problem gamblers. Third, the present sample mainly comprised females while the prevalence rates of disordered gambling are higher among males (e.g., Ciccarelli et al. 2019a, b; Nigro et al. 2018). Moreover, the internal consistency of the subscale 'perceived inability to stop gambling' was low. Finally, since the present sample mainly comprised sport bettors (see Table 1), the results may not apply to all types of problem gamblers. Future studies addressing the specific metacognitive impairment in problem gambling are encouraged.

Despite the limitations, the present study provided—for the first time—insight into the interrelationships between poor mentalization, emotion dysregulation, and cognitive distortions that together contribute to problematic gambling behavior in adolescence. It may be that gamblers (having difficulties in managing negative emotions and experienced as overwhelming) engaged in impulsive behaviors such as gambling in order to dampen the emotional arousal. Emotional dysregulation is associated with mentalization impairments that makes difficult for gamblers to understand their own mental states, especially when powerful emotions arise. As the engagement in gambling increases, cognitive distortions concerning personal ability and control over gambling outcomes appear, strengthening gambling involvement levels (Blaszczynski and Nower 2002) and increasingly contributing to blurring of the ability to mentalize and to consider the negative consequences of gambling.

Interventions focused on emotion regulation could be effective in avoiding the use of maladaptive ways of managing emotions, such as substance or behavioral addictions. This type of training could help individuals to become more aware of their internal states and to learn the strategies to adaptively manage emotions. Moreover, recent studies have demonstrated that prevention programs focused on cognitive distortions (and delivered in school settings) are effective in reducing misconceptions about gambling and the time spent in gambling activities. These studies also demonstrated the stability of the training effects over time and their potential to offset the trajectory toward disorder (Calado et al. 2020; Donati et al. 2018).

Along with interventions on cognitive distortions, a metacognitive intervention could also help adolescents to reduce their gambling involvement. As suggested by Toneatto et al. (2007), “learning to relate differently to gambling cognitions may be as important as, if not more important than, challenging the specific contents of the thoughts” (p. 94).
